# Palliative sedation challenging the professional competency of health care providers and staff: a qualitative focus group and personal written narrative study

**DOI:** 10.1186/s12904-017-0198-8

**Published:** 2017-04-11

**Authors:** Danièle Leboul, Régis Aubry, Jean-Michel Peter, Victor Royer, Jean-François Richard, Frédéric Guirimand

**Affiliations:** 1 Pôle Recherche SPES « Soins Palliatifs En Société », Maison Médicale Jeanne Garnier, 106 avenue Emile Zola, 75015 Paris, France; 2 INSERM CIC1431, Centre Hospitalier Universitaire de Besançon, 2 boulevard Fleming, 25030 Besançon, France; 3 CERLIS, UMR CNRS 8070, Université Paris Cité Sorbonne, 45, rue des Saints-Pères, Paris cedex 06, France; 4 Institut de Recherche Interdisciplinaire sur les Enjeux Sociaux (UMR 8156), Ecole des Hautes Etudes en Sciences Sociales, 190-198 avenue de France, 75244 Paris cedex 13, France

**Keywords:** Palliative sedation, Professional competency, Workplace suffering, Palliative care, Midazolam

## Abstract

**Background:**

Despite recent advances in palliative medicine, sedating a terminally ill patient is regarded as an indispensable treatment to manage unbearable suffering. With the prospect of widespread use of palliative sedation, the feelings and representations of health care providers and staff (carers) regarding sedation must be carefully explored if we are to gain a better understanding of its impact and potential pitfalls. The objective of the study was to provide a comprehensive description of the opinions of carers about the use of sedation practices in palliative care units (PCU), which have become a focus of public attention following changes in legislation.

**Methods:**

Data were collected using a qualitative study involving multi-professional focus groups with health care providers and staff as well as personal narratives written by physicians and paramedical staff. A total of 35 medical and paramedical providers volunteered to participate in focus group discussions in three Palliative Care Units in two French hospitals and to write personal narratives.

**Results:**

Health care provider and staff opinions had to do with their professional stance and competencies when using midazolam and practicing sedation in palliative care. They expressed uncertainty regarding three aspects of the comprehensive care: biomedical rigour of diagnosis and therapeutics, quality of the patient/provider relationship and care to be provided. Focusing on the sedative effect of midazolam and continuous sedation until death, the interviewed health care providers examined the basics of their professional competency as well as the key role played by the health care team in terms of providing support and minimizing workplace suffering. Nurses were subject to the greatest misgivings about their work when they were called upon to sedate patients.

**Conclusions:**

The uncertainty experienced by the carers with regard to the medical, psychosocial and ethical justification for sedation is a source of psychological burden and moral distress, and it has proved to be a major source of suffering in the workplace. Lastly, the study shows the uncertainty can have the positive effect of prompting the care team to devise ways to deal with it.

## Background

Despite recent advances in palliative medicine, sedating a terminally ill patient is regarded as an indispensable treatment to manage refractory symptoms such as severe agitated delirium or asphyxial dyspnoea and to relieve intractable distress [1–3]. Palliative sedation can be used for a short period (intermittent sedation) or continuously until death and its depth varies from a lower level of consciousness to unconsciousness. These various characteristics partly explain the wide-ranging prevalence of this last resort treatment reported in the literature (1–88%) [1]; and the practice may in fact be under-reported [3]. The biomedical and ethical aspects of palliative sedation are nevertheless widely debated [4–8] and best practice guidelines have been adopted [9–15]. In France, since 2009, national guidelines have been available [16] and recently, the right for the patient to ask a deep continuous sedation until death under certain conditions is supported by the law [17].

Many studies address concerns among health care providers – nurses and physicians – and staff, who report experiencing uneasiness, moral distress and emotional burden [14, 18–24] over palliative sedation. Morita, in his seminal study (2004), identifies the main factors associated with emotional burden in nurses [25]. These factors include acquisition of the necessary skills and work organisation. However, the questionnaire-based survey was unable to provide a deeper understanding of these issues. Despite these concerns, palliative sedation appears to have been spreading in recent years and will involve an increasing number of health care providers in hospitals, medical centres and home health care settings going forward [26, 27]. With the prospect of widespread use of end-of-life palliative sedation, the feelings and representations of health care providers and staff regarding sedation must be carefully explored if we are to gain a better understanding of its impact and potential pitfalls. The objective of this study was to provide a comprehensive description of the opinions of physicians and health care staff about the use of midazolam and sedation practices in French palliative care units, which have become a focus of public attention following changes in legislation.

## Methods

### Methodological approach

The study employed a qualitative methodology based on a comprehensive approach and an inductive conceptualisation process, according to grounded theory approach [28]. The qualitative study was undertaken to explore the perspectives of health care providers and staff regarding the use of midazolam and the practice of end-of-life sedation and the ways in which they were affected by the processes underlying their experiences, perceptions and representations.

### Study design and data collection

The study was conducted by a multidisciplinary research team composed of three palliative care physicians, a psychologist, a sociologist and an ethnologist all of whom were familiar with qualitative methods. Health care providers and staff were informed of the study’s aims and asked if they would be willing to participate. Data was collected from March 2012 to December 2012 through focus group interviews and personal written narratives. Three multi-professional focus group discussions (two three-hour sessions) took place in three settings: two Palliative Care Units (PCU) in a PC hospital in Paris and one PCU in a university hospital in Besançon. A total of 28 medical and paramedical providers participated. Each group was composed as shown in Table 1. The discussion was introduced by the following sentence: “midazolam is a drug used in palliative care. Everybody is aware about it and knows something about its use. From your personal experiences, we are going to confront the opinions about its use and share the purposes for which midazolam was used”. The focus group guide included the following topics: the terms used: which word to say what?, the description of the clinical context in which the question of using midazolam arisen, the knowledge about midazolam, how healthcare providers and staff analysed and interpreted the effects of the medication, the implementation of the injection, the ethical and moral implications of the practice, the team strategy. Data saturation was reached at the third focus group.

**Table 1:** Composition of focus groups

	Location	Profession	Age	Lenght of service	Number
Focus Group	PCU 1 Paris	physician	50–59	8.5 years	3
			50–59	16.5 years	5
		nurse	30–39	2 years	1
			40–49	2 years	8
			40–49	4.5 years	9
			20–29	4 years	10
		head-nurse	40–49	8 years	4
		assistant nurse	50–59	2.5 years	7
		psychologist	40–49	4 years	6
		volunteer	60–69	3 years	2
	PCU 2 Paris	physician	60–69	6 years	4
			40–49	2 years	7
		nurse	30–39	5 years	3
			40–49	6 months	6
		assistant nurse	50–59	8 years	1
			40–49	3 years	9
		physiotherapist	50–59	3.5 years	5
		psychomotor therapist	30–39	6 months	8
		volunteer	60–69	12 years	2
	PCU 3 Besançon	physician	40–49	2.5 years	9
		nurse	40–49	7 years	4
			40–49	5 years	6
		assistant nurse	50–59	2 years	1
			50–59	9 years	7
			30–39	1.5 years	3
			50–59	10 years	8
		psychologist	50–59	2 years	5
		physiotherapist	50–59	4 years	2

All health care professional providers and staff of the 3 PCU were asked to write personal narrative focussing on how they were concerned by the use of midazolam according to a detailed instruction. Fourteen personal narratives written by 11 physicians and health care staff were collected (Table 2).

**Table 2:** Written narrative contributors

	Location	Profession	Age	Lenght of service
Written narratives	Paris	nurse	20–29	6 months
		nurse	40–49	5 years
		nurse^a^	30–39	5 years
		nurse	30–39	2.5 years
		nurse	40–49	8 years
		nurse	20–29	2 years
		physician	50–59	7 years
		physician	50–59	10 years
	Besançon	physiotherapist^a^	50–59	4 years
		physician^a^	40–49	2.5 years
		assistant nurse^a^	30–39	1.5 years

^a^Participant to a focus group too

At least 35 health professionals and three PC teams from three PCUs volunteered to participate. There was no hierarchical relationship between the health care providers and the researchers.

### Data analysis

A thematic analysis of the full transcripts of the focus group discussions was carrying on in real time. The focus group discussions and narratives were read, reread and coded at a first level by two researchers, a doctor and a psychologist, which working independently from one another. A constant cross comparison enabled the researchers to agree about the attribution of codes to verbatim, then to pool codes in categories and finally to draw up a topic tree. This topic tree was discussed by the multidisciplinary research team. An agreement was reached in identifying four major themes.

This study was approved by the Institutional Review Boards of the institutions ; it was conducted and reported taking into account the consolidated criteria for reporting qualitative studies [29].

## Results

The multidisciplinary analysis of the data highlighted health care provider and staff opinions regarding their professional stance and competencies when using midazolam and practicing sedation in palliative care. They expressed uncertainty regarding three aspects of comprehensive care: biomedical rigour, quality of the patient/provider relationship and care to be provided. Focusing on the sedative effect of midazolam and continuous sedation until death, the interviewed health care providers examined the basics of their professional competency as well as the key role played by the health care team in terms of providing support and minimizing workplace suffering.

### Uncertainty concerning diagnostic and therapeutic rigour

Health care providers and staff were aware of the recommendations regarding sedation and the use of midazolam to relieve painful and refractory symptoms – confusion, major anxiety, dyspnoea, intractable pain, psychological distress and existential suffering [16, 30]. To cope with emergency situations involving respiratory distress or cataclysmic haemorrhage, physicians and nurses had drawn up sedation protocols and standing orders for medication administration. Yet, as our results show, in practice they found it difficult to follow these protocols and administer the medication. The following statements illustrate their difficulty in determining when midazolam was indicated and deciding the proper time of administration.

#### Doubts regarding symptom identification

The health care providers described their difficulty in interpreting clinical signs to clearly identify a symptom.

*Patients sometimes grimace, if you could call it that… I don’t think this is anxiety, but just one of the changes that take place at the end of life. Nurse’s assistant 7; focus group (FG) 3.*

*When we interpret these signs as anxiety in the patient, we are expressing our own anxiety. It is a very difficult, subjective call. Pain can be sensed and observed, but anxiety… Physician 5;FG1.*

*I hold back in administering these medications despite the standing order because, again, it is difficult to predict respiratory distress. I prefer to interpret respiratory distress when it can be observed. We run the risk of increasing patient suffering when the patient is about to experience respiratory distress, but at the same time there are so many risks of misinterpretation that we need to have clearly defined criteria to go on and not mere anticipation. We need evidence. Physician; 5;FG1.*

#### Influence of the symptom on the health care provider’s affective and emotional response

Health care providers fear that their judgment will be influenced by the effect of the patient’s symptom on their own emotions, impairing their ability to analyse the situation and make decisions.

*Agitation is upsetting. Physician 9; FG3.*

*… And I think it frightens some health care staff. Nurse’s Assistant 7; FG3.*

*The question is, how far can you go when you see agitation? I know I find it difficult to cope with agitated patients, I would prefer for them to die peacefully, I know that. I feel a lot more comfortable when they are calm. Nurse’s Assistant 1; FG3.*

#### Indecision about the benefits of the sedative effect of midazolam

They described a critical interpretation of the consequences of sedation, contrasting the negative and positive effects of the medication:

Some saw the sedative as preventing the patient from any form of expression and communication and as destroying the meaning of the last moments of his/her life.

*Mr X held out his hand, I took it, we looked at each other for several minutes, it was great. That is what I want to experience. And if he had had a dose of midazolam, tick, tick, tick… I think he was happy. Nurse’s Assistant 7; FG3*

*I have the impression that if you give them midazolam at that time, they will not say what they have to say, at that time. Nurse; Personal narrative (PN).*

Others saw the sedative as having the beneficial effect of calming the patient and preserving his/her ability to communicate.

*I think that midazolam enables them to experience time, to tame a small amount of time, to be more peacefully with their family. I see midazolam as a beneficial alternative for the patient. Night shift Nurse’s Assistant 1; FG2*

*I think that when you are suffering to that extent, you cannot experience anything else. From what I have seen of midazolam, it provides relief from unbearable anxiety and distress. Physiotherapist 5; FG2*

#### Ambivalence about the “power” of midazolam

Some health care providers and staff also felt that midazolam was sometimes unable to treat the symptom. Basing their comments on experience, they were highly ambivalent about its effectiveness.

*My attitude toward midazolam has changed. I have a somewhat positive impression, with some ambivalence. At one time I thought it was an all-powerful sedative – the medication allowing us to provide tremendous relief when everything else had failed. And then in rapid succession I had several cases in which midazolam did not produce the expected effect. I felt in a way that midazolam had let me down. Physician 7; FG2.*

The subjective filter in interpreting the symptom and doubts about the advisability and effectiveness of midazolam caused providers and staff to question the refractory nature of the symptom which would have forced them to sedate the patient, and also to question the indication for sedation. Coupled with both the uncertainty on the clinical reality and the appropriateness of indication for sedation, the healthcare providers’ anticipation of the effects of sedation made them feel confused and powerless in dealing with the therapeutic decision making of either to sedate the patient or not.

### Uncertainty as to the quality of the patient/provider relationship

The patient/provider relationship magnified the uncertainty regarding how to proceed since it increased the care provider’s ambivalence about feeling all-powerful, exercising control and having the power inherent in knowledge.

#### Reversal of positions of knowledge

Care providers reported feeling at a loss when faced with a patient’s request for sedation in the absence of a symptom warranting it. The patient who asks to sleep is in fact expressing what he/she does not want: he/she does not want to be uncomfortable.

*Yesterday evening the patient said to me, “Can’t you give me a little something so I can sleep like I did the other day?” Night shift Nurse 6; FG2.*

He did not want to be conscious because consciousness was painful or unbearable.

*I had the impression I had become a midazolam pump. The patient refused to see the psychologist or the psychomotor therapist. Once you give in, every four hours the patient says, “Give me the magic potion that will let me sleep” and I find that very unpleasant. Nurse 8; FG1*

The request for sedation, no longer based on a clear-cut symptom of discomfort but rather on psychological suffering or the anticipation of a symptom, reversed the positions of the care provider and the patient or the patient’s family members with respect to knowledge and competency. The care provider felt the decision was no longer in his hands.

*There is the patient who says “I want to sleep,” and then there is the health care provider who says for example “I will use the standing order because he is in pain,” and I am the one who decides to induce sleep because I think that is what he wants. Psychomotor therapist; 8; FG2.*

Some care providers were afraid of causing addiction or of embarking on a path towards indiscriminate and unwarranted use of midazolam.

#### Torn between complying with patient requests and resisting patient manipulation

Other interconnected factors affected the reticence of care providers to meet the patient’s requests. The first was that care providers spontaneously interpreted the request for sedation as a request for continuous sedation until death.

*Right now, 99% of the requests for sedation are for CSD. Nurse’s Assistant 9; FG2.*

*I don’t get requests for 48-hour temporary sedation any more. People don’t want it. They say: “I want to sleep until the end,” and that is what we negotiate. Physician 7; FG2.*

The second factor is that when the patient is no longer able to communicate, he/she can no longer confirm the benefits of the care received. Indeed, when a person is put to sleep his or her body becomes an inert object that can be manipulated at will. In the absence of responsiveness, the health care provider felt unable to assess the treatment’s effectiveness and to ethically validate its purpose. As a result, there is a perceived risk of doing harm. In other words, the health care provider is no longer in a position to say “I know what is good for you,” and has no evaluation criteria enabling him to distinguish between benefit and harm.

When in doubt, health care providers feared being manipulated and were worried about the negative judgment of the community, which included end-of-life volunteers.

*Family members expressed it this way: “I can’t ask you for euthanasia because it is not legal in France, so I ask you to give him something to let him sleep!” That was the wife. Physician 7; FG2.*

*The volunteers say, “Ah, yes, they are drugged out,” and believe it might be for the convenience of the staff. I had given an intermediate dose for the comfort of the family. There is a lot of comfort involved – the comfort of the patient, the family and the health care staff. Physician 7; FG2.*

*I think that the family did not have the same values that we did. So we acted in a way that satisfied the family, but for a purpose that was radically different. For the family, the action led to death and for us the action was meant to provide relief. And therefore, what the family can say about it… Physician 7; FG2.*

#### Torn between providing pain relief and sedating

*Ultimately, I don’t think you shorten the patient’s life, but you do shorten his relationships. Physician 3; FG1*

All groups perceived the interruption in communication induced by sedation as a failure to achieve what they considered the ideal solution, in which the patient would remain in contact with others until the end. All groups saw the last moments of life as fundamentally meaningful. Beyond these representations, the perception of failure in the relationship was due to the fact that care providers were no longer able to negotiate with the patient. The patient could no longer cooperate in relieving his distress and optimising his “comfort”. He was no longer able to provide informed consent. A profound change had taken place in the patient/provider relationship, which had been established on the basis of agreement and mutual understanding as a relationship based on the caregiver’s empathy, enabling the caregiver to assess the use and effectiveness of the medication on the patient’s feelings, bodily sensations and psychological state as expressed by the patient. The cessation of this relationship increased the provider’s uncertainty as to the suitability of the care in meeting the patient’s needs, since both the patient and other providers were now unable to assess the purpose and quality of the patient’s response in a continuous process of adjustment and evaluation of the treatment’s effectiveness.

### Uncertainty regarding the care to be provided

In addition to the loss of communication in the patient/provider relationship, health care providers expressed three further concerns about the therapeutic effects of sedation.

#### Does putting people to sleep automatically give them relief?

*Despite the midazolam, the patient continued to moan, but in her sleep. Nurse’s Assistant; PN.*

One question came up time and again: What does the patient experience when sedated? What of his psychological suffering? Can the patient continue to suffer?

This question related to the depth of sedation and to the dose of midazolam administered. Despite their clinical experience, health care providers were unable to determine whether sleep and relief coincided.

Based on the usual representations of sleep as a repair mechanism, night shift staff had a different perspective from that of their day shift colleagues and some saw the interruption of communication in a different light. They approved the use of midazolam whatever its effects and believed that reduced consciousness resulted in relief. They saw it as making the patient comfortable and also calming the environment.

*Of course, one consequence is that putting the patient to sleep means that he/she can no longer communicate. That is clear. But patients do not belong to us and I am not sad when they can’t talk to us. It is not about me or about whether they can talk to me. It is about making them as comfortable as possible. If it takes midazolam and sedation to achieve that, why not? Night shift Nurse 6; FG2*

#### Are we hastening the patient’s death?

*Yesterday morning we had a gentleman who was very anxious. We weren’t able to make him comfortable, so we gave him a shot of midazolam and for a moment we thought to ourselves, “When the patient is at the end of life, o.k., but that is no reason to make him die!” We had a moment of doubt. Nurse’s Assistant 1; FG3.*

The fear of accelerating or even causing the death of the patient was widespread among care providers. This was due to the implicit equation Midazolam = Sedation = Accelerated death, which in its most radical form is tantamount to Midazolam = Sedation = Killing the patient = Euthanasia. This fear was a major issue and a burden for care providers, even in highly managed situations in which the team had discussed the decision to sedate, the arguments for and against it had been debated and the care providers had agreed with the decision despite their differing views regarding sedation and its timing.

Nurses stressed their difficulty in accepting responsibility for the “physical act” of injecting midazolam and attempted to articulate the intense stress they felt. The severe anxiety they experienced at the moment they administered the sedation was due to the loss of control they had experienced in previous situations of the same type. When they were called on to perform the injection, they were afraid of making a mistake in calculating the dose, which might have fatal consequences.

*If I make a mistake in drawing up the syringe, it can have consequences that can even be fatal. And so I have to be extra careful and make sure I don’t do anything wrong. Nurse 1; FG1.*

They were alarmed by the rapid effect of the medication, describing it as “*impressive*” and saying it “*frightened*” them.

*It doesn’t happen often, but when it does it makes an impression! When I give midazolam to a patient who is exhausted and the patient reacts immediately, it is truly impressive. Nurse 9; FG1*

*“And I even thought at first that she was dying. It is very, very fast acting.” Psychomotor therapist; 8; FG2.*

They were afraid of being responsible for the patient’s death.

*When I do something, when I take action, I find it hard to tell myself that it was not what I did that led to the situation that follows. Nurse 1; FG1*

*You always have the thought that when you have done something and the patient dies shortly afterwards it was you who killed him. Head nurse 4; FG1*

The physical act of injecting midazolam provoked deadly representations and destroyed the rational connection between the intent and the act, the all-important factor in the decision-making process that provides legitimacy for the action.

*But there is often a big difference between thinking things and experiencing them. We are in relative agreement about the decision to sedate because the patient is experiencing major existential suffering. But when the time comes to inject the medication, things seem less clear-cut. Nurse 4; FG3*

*What am I doing that I don’t want to do? Nurse 1; FG1*

Paramedical staff such as nurse’s assistants and physiotherapists, who are not called on the inject midazolam, did not experience the distress described by nurses. They remained focused on the goal of providing relief for the patient.

*For me, it gives the patient psychological relief and removes stress for both the patient and for us. It is painful to see the patient suffering do nothing. Afterwards, I don’t actually see the death. Nurse’s Assistant 1; FG2*

#### What is the meaning of patient care when the patient is under continuous sedation until death?

Health care providers clearly saw an analogy between the terminal phase of the patient receiving continuous sedation until death and actual death. They followed common practice designed to support the patient, while having to confirm the rationale for doing so under the circumstances.

*I had a case of continuous sedation until death. I was even glad I was there when the patient died because I had cared for her in recent days. We were there for her, supporting her until the end. That was reassuring for the team. But she was sedated… We talked to her until the end, and she was calm when she died. Nurse’s Assistant 3; FG3.*

To explain the meaning of supportive care in their experience, they described how watching the patient die raised existential questions.

*The terminal stage, when the patient is going to die, is a special moment when the family is often in the room and time is suspended. And this creates a special approach to death in each of us, whereas before, we were all focused on caring for the patient. Nurse 6; FG3*

Most health care providers said that being with the patient gave meaning to the work they accomplished.

*Yes, it gives meaning to what we have done. That’s important. Being there is meaningful, to me. Nurse’s Assistant 1; FG3.*

*It is the culmination of everything that has been done to support the patient and the patient’s relatives. Physician 9; FG3.*

But some questioned this certainty.

*Ultimately, have you done it for yourself or for the patient? You may be imagining it. Nurse’s Assistant 1; FG3.*

*We talk about the expectations of everyone involved! What is the best, or rather the least bad, way to die? Physician 9; FG3.*

### Working with uncertainty in a clinical setting: inventive teamwork

Health care professionals sought ways to ensure that midazolam was indicated and not being overprescribed, on the one hand, and to lighten their own burden of discomfort and suffering, on the other.

They engaged in team discussion about the issues and made the multidisciplinary team a requirement to ensure high-quality care.

*I tell myself: the great thing is for us – who represent different professional categories and different age groups – to put our heads together. That is what will probably enable us to arrive at what is best for the patient. Psychomotor Therapist 8; FG2.*

Team deliberation was seen in a positive light. It covered the meaning of clinical practice and supported the creation of expertise in the prudent use of sedation and changes in the way work is organised.

#### Deliberation at all stages of the process

Before: *We need to take time to understand and assess the situation, since we do not know the patient. This is why we do not immediately meet a request for sedation. But we do explain to the patient that we could possibly meet it, that we need about two weeks or even three weeks to understand the reasons for the request. Physician 5; FG1.*

During: *It is so easy to say: midazolam will probably work. But what we should actually be doing is taking time to think about it and ask whether some other approach would be better. Night shift Nurse 6; FG2.*

After: *We try to take time to carefully examine the situation at a time when there is no emergency, when we are more in control of our emotions. Nurse, PN*

#### Expertise in the prudent use of sedation

Health care providers sought to avoid what they called the “overuse” of midazolam.

*I work the day shift and I think there are times when we are hasty in administering midazolam. This only happens occasionally, but it can happen fairly regularly, depending on the team, on the patient and on the workload. And sometimes we give midazolam too readily. Night shift Nurse 3; FG2*

They were careful in administering the midazolam prescription appropriately. They paid attention to the way the prescription was worded.

*We know quite well that midazolam is given for insomnia, it’s in the prescription. It is not intended as a medication to induce sleep. Night shift Nurse 6; FG2.*

They developed a cautious, wait-and-see approach to the techniques and the patient/provider relationship, based on the knowledge they had gained from experience and on observing the patient in his or her context.

*Patients need to get used to the staff. Every time a person comes in, I compare the situation to a jar of water and sand that has been shaken: you have to wait for things to settle, you have to give them time to settle in. You don’t give them midazolam right away; you wait awhile. Nurse’s Assistant 9; FG2.*

*When I have an agitated patient, even if midazolam is available, I ask every patient the same question: “Are you in pain? Do you want us to do something?” Certainly you don’t administer midazolam as you would Zopiclone that has been prescribed. I don’t give it the same way. You take time. You don’t give midazolam to every agitated patient. Night shift Nurse 6; FG2*

They used interventions that do not require medication and involve being with the patient, attempting to distract him/her to relieve anxiety or suffering (talking to the patient, holding his hand, having him listen to music, etc.) and getting all staff member (psychomotor therapist, psychologist, sophrologist) competencies and end-of-life volunteers to participate.

*The patient was trying to cling to me, we struggled, I took her in my arms and cradled her with songs and she relaxed. Psychomotor Therapist 8; FG2*

They based themselves on each other’s experience in weighing their decisions and taking action.

*The team had very carefully prepared the sedation; we talked it over as a team and decided to administer it. Physician 7; FG2*

*I know that I base my decisions partly on the views of my co-worker. I think I can use her experience. I think we need to share our experience. That is what is called teamwork. Nurse 4; FG3.*

#### The work organisation process

In this way, they developed tacit or explicit rules governing their work.

*What we would like to try to do is to let other care providers know what would be a good approach… and pass on our tools, you see. Assistant nurse 9; FG2*

The rules changed the way the work was organised: a two-person team to provide nursing care, not being alone when injecting midazolam during sedation, taking part as a team in giving report to pass on information several times a day at fixed times. Report covered precise medical information and detailed descriptions of behaviour and the environment, to ensure continuity of care.

*Fortunately we have all these opportunities to give report, and fortunately we document what we do because when the verbal report is short, I have the written report from previous nights to make sure I have got it right. Night shift nurse 6; FG2*

#### Team strategies to combat suffering

Discussing care, making a joint effort to build competency and working together helped relieve staff burden but could not do away with it altogether. To reduce the distress, fear or guilt caused by uncertainty and stressful situations, health care providers supported each other with reassurance and friendly banter.

*Obviously, and fortunately, there is always someone to tell us, “Don’t worry, the patient was weak, you weren’t the one to kill him,” and so on. Nurse 1; FG1*

*We tease each other: “No way, you’re the nurse, you killed him!” Nurse 1; FG1.*

But they also shared a strategy of avoiding the reality of sedation by not mentioning it or by using euphemisms such as enabling patients to sleep.

## Discussion

Sedating a patient in palliative care is not routine practice. It engages care providers in a holistic and complex approach to patient suffering that often leaves them uncertain about the best way to respond to it [31, 32]. Our study brings out a major finding: the destabilisation of health care providers’ confidence in their own competence. The psychological and emotional confusion that palliative sedation can induce in care providers is a manifestation of the suffering caused by the destabilisation of their work in providing care and support. This workplace suffering is fundamentally due to their doubts, at several levels, about their own professional competence. Health care providers doubt their ability to reason and build a clinical action plan; they experience difficulty in analysing, defining and interpreting the patient’s symptoms, in determining whether the symptoms are refractory and in accepting that they are unbearable for the patient. The absence of an agreed definition of the term “refractory” may be a contributing factor [2]. As a consequence, they hesitate to use the indicated treatment in cases of major physical or existential suffering, especially since they fear that the medication may not be effective but also fear its sedative effects [5, 24, 33]. This uncertainty compounds the doubts they express regarding the patient/provider relationship. Their ability to maintain and further build a relationship with the patient in order to provide support and relieve suffering depends on the patient’s being able to articulate pain and anxiety through words and body language, attitude or symptoms. When the patient expresses suffering but the body shows no signs of it, or when the patient can no longer communicate his suffering in words or body language, the patient/provider relationship is shaken for lack of support. When the patient is sedated, his/her reduced consciousness, inability to communicate and sleeping state leave health care providers at a loss to know whether they are providing appropriate care. They are forced to make decisions on behalf of the patient, and doubt their own ability to assess the quality of the care provided. With no feedback to go on, how are they to judge whether the result meets the goal of relieving the patient’s suffering, which is the focus of the decision-making process? How can they be sure that the steps taken to achieve that goal are the right ones? The uncertainty experienced by health care providers about the legitimacy of their action becomes ingrained:

- in a lack of recognition of its usefulness
- in doubts about compliance with rules – not only those of best practice but also those generally accepted as the “gold standard” of palliative care.

All health care providers experience these doubts, but the literature shows that emotional burden varies from one professional category to another [23, 31, 34, 35]. Our study confirmed previous studies showing that nurses, who play an important role in sedation, appear to be the group most vulnerable to doubt during sedation [22]. Following the discussion and the medical decision to sedate, they are the ones involved in its “ultimate” implementation: preparation and injection of the medication. This action in itself amplifies doubt and causes nurses to feel it more acutely. Their involvement in the act of injecting the sedative, their physical closeness to the patient and their simultaneous perception of the patient’s response to the medication are factors determining the burden caused by perceived uncertainty [36]. In practice, the representation of uncontrolled, potentially harmful consequences of the sedative often overrides the representation of its benefits, i.e. its ability to relieve the refractory symptom and the unbearable suffering. This results in a clash between their technical expertise (preparing and injecting the sedative) and their expertise in providing complex care. Nurses may see this as a challenge to their professional identity and their integrity. The gravity of the act undermines the goal of relieving suffering which constituted the ethical basis for the decision to sedate. The intent to do no harm and to avoid hastening the death of the patient is not enough to reassure nurses or relieve them of feelings of guilt [37]. They find it difficult to accept their ambivalence toward sedation – the tension between the reasoned approach to the decision based on the intention to provide relief and the fear of harmful consequences that the power of the act of sedation generates in their minds [38, 39]. This tension should be examined from an ethics perspective, by looking at intention to treat principles in care provision and in clinical practice [40–43] and at the fact that although the decision is made by the team, its implementation involves the conscience of the individual nurse or physician carrying it out [6, 44–46].

Examining the relationship between intent and consequence requires an in-depth consideration of the relationship between the team and the individual during decision making and implementation, based on an analysis of current practice [20, 47]. The health care team plays a crucial role in managing uncertainty, since it enables its members to express their feelings in words and articulate what is being done [20, 47]. Teamwork helps reduce individual distress by clarifying the course of action, providing an opportunity for dialogue and organisation and offering professional recognition and support [48]. In addition to extending psychological support to health care providers [22], teamwork enables providers to build cooperation and devise ways to arrive at agreed rules governing their work. Teamwork thereby makes it possible for providers to reconfigure their approach to their professional competence in the sedation situations that constitute a burden for them [49]. Teamwork supports the endeavour to acquire expertise and achieve excellence [32, 50]. Taking uncertainty on board as a clinical reality to be dealt with in the practice of sedation initiates a process of emancipation, by definition collective, leading to new, better ways of living together.

It is interesting to note that the beneficial results of managing uncertainty belong to similar domains to those covering the main results of Moral Case Deliberation (MCD); in particular: enhanced emotional support ; enhanced collaboration ; impact of organisational level ; concrete results [51]. The ethic complexity of situations of sedation and the importance of team work both at the moment of deliberation and during the reflexive analysis of the action, would need better structuration of the collective exchange. This could be obtained according to the process MCD, allowing an evaluation with the aid of instruments such as Euro-MCD [51, 52]. Based on experience of health care providers in managing uncertainty, a training program to MCD with a mix of theory and practice would contribute to their training [53].

### Strengths and limitations of this study

The construction and rigorous implementation of the qualitative methodology supports the scientific robustness of the results with respect to the following criteria:

- validity: triangulation of methods used to collect data, interdisciplinary cooperation of researchers, cross-comparison of results;
- reliability: results strictly derived from the content of the text-based data; and
- transferability: the context of the study was well-defined; the population was characteristic of palliative care teams, with reference to values and codified clinical practices in several settings with similar organisational features [54, 55]. However – and herein lies the limit of the study – although a case can be made for the validity of the results in Palliative Care Units, these results cannot be extrapolated to other health care facilities providing palliative patient care [18, 34, 47], much less to different countries. These results regarding palliative care provider attitudes towards sedation and the impact of sedation practice on their perception of their own professional competence would have to be tested in different cultural and legislative settings, for example by carrying out comparative studies at European level [18, 34, 47].

## Conclusions

Our study contributes to a better understanding of the opinions and perceptions of health care providers working in Palliative Care Units. It highlights the uncertainty experienced by these providers with regard to the medical, psychosocial and ethical justification for sedation. This uncertainty affecting them is a source of psychological burden and moral distress, and in addition it has proved to be a major source of suffering in the workplace. Uncertainty with respect to the three levels of expertise in palliative care causes health care providers to doubt their own professional competence and ability to perform their duties.

Lastly, this study shows how the uncertainty caused by the complexities of sedation can have the positive effect of prompting the care team to devise ways to deal with it. Uncertainty motivates the team to think about, debate and clarify the action taken. By sharpening their sense of responsibility, uncertainty gives care providers an incentive to seek new expertise and to make changes in the work organisation of the multidisciplinary care team.

## Abbreviations

PC: Palliative care
PCU: Palliative care unit
